# Supplementary material for: A self-sustainable wearable multi-modular E-textile bioenergy microgrid system
Source: Nat Commun. 2021 Mar 9;12:1542. doi: 10.1038/s41467-021-21701-7 (PMC7943583; doi:10.1038/s41467-021-21701-7)
Supplement: Supplementary file 1 — Supplementary Information [file 41467_2021_21701_MOESM1_ESM.pdf]

A Self-Sustainable Wearable Multi-Modular E-Textile Bioenergy Microgrid System

Lu Yin[1], Kyeong Nam Kim[1], Jian Lv[1], Farshad Tehrani[1], Muyang Lin[1], Zuzeng Lin[1], Jong-Min Moon[1], Jessica Ma[1], Jialu Yu[1], Sheng Xu[1], Joseph Wang[1]*

[1]Department of NanoEngineering, Center of Wearable Sensors, University of California San Diego, La Jolla, California 92093, USA.

These authors contributed equally to this work: Lu Yin, Kyeong Nam Kim, Jian Lv

*Correspondence to: josephwang@ucsd.edu

Contents

Supplementary Note 1: Fabrication, Optimization, and Characterization of the Triboelectric Generators (TEGs) 2

Supplementary Note 2: Fabrication, Characterization, and Optimization of the Biofuel Cells (BFCs) 6

Supplementary Note 3: Fabrication and Characterization of the Supercapacitors (SCs) 10

Supplementary Note 4: Characterization and Discussions of the In-Vitro and On-body Operations of the Integrated Bioenergy Harvesting-Storage Modules 14

Supplementary Note 5: Design, Fabrication, and Characterization of The Wearable Applications in the Microgrid 18

Supplementary Figures 26

Supplementary Movies 59

Supplementary References 60

Supplementary Note 1: Fabrication, Optimization, and Characterization of the Triboelectric Generators (TEGs)

1. Design, Fabrication, and optimization of TEGs

The fabrication of the TEG modules is based on screen-printing of formulated polymer composite inks. The TEG module is comprised of two parts: the stator, which includes the positively charged ethylcellulose-polyurethane (EC-PU) composite and the interdigitated flexible silver current collectors; and the mover, which includes the negatively charged PTFE and fluorocopolymer binder. A layer of SEBS resin and EC-PU ink were printed onto the stator and the mover, respectively, as the lining to smoothen the surface. The flexible silver ink was then printed onto the SEBS lining in two sets of interdigitated electrodes. The PTFE powder was mixed with the T-70 fluorocopolymer which was comprised of vinylidene fluoride, tetrafluoroethylene, and hexafluoropropylene were printed onto the mover as the negatively charged electrode. A sheet of EC-PU was cast onto the current collectors as the positively charged stator. The fabrication process is illustrated in **Supplementary Fig. 1**.

The textile-based TEG module can generate electricity from the combination of the electrification of two contacting layers during the sliding motions and the electrostatic induction to the back electrodes. The electrification takes place when the PTFE mover slides across the EC-PU film of the stator, where electrons are transferred at the interface from the PTFE composite to the EC-PU composites due to their difference in the electron affinity, allowing the PTFE electrodes to be negatively charged and the EC-PU to be positively charged temporarily. This effect is combined with the electrostatic induction between the EC-PU and the silver current collector on the stator. When the PTFE mover slides across the stator surface, the parallel PTFE patterns momentarily align with one of the two sets of the interdigitated silver current collectors below the EC-PU layer alternatively. As a result, the current flows from one set of the current collectors and back, alternating the polarity between the two sets of electrodes between positive and negative, and thus forming an alternating current[1–4].

To measure the performance of the TEG during the sliding, the two electrodes on the stator were directly connected to an oscilloscope, and their open-circuit voltage (V_{OC}) and short-circuit current (I_{SC}) were recorded. To optimize the design of the TEG module, patterns with different

grating density were fabricated and their maximum peak voltage when sliding at 3 Hz was recorded, as shown in **Supplementary Fig. 2**. Although the output voltage dropped slightly as the number of segments increases, the peak current increased dramatically with the segments, indicating an increased amount of charge transfer as the grating density. The output reached a maximum of 130 µA with a corresponding peak voltage of 150 V with the 64-segment design. The drop in voltage can be attributed to the decreased pitch between individual lines, which in turn facilitated the increase in the oscillation frequency and led to a higher peak current. The raw voltage and current output of the TEG module within one stride are illustrated in **Supplementary Fig. 3**. Overall, the total transferred charge of the TEG module increased with the grating frequency, thus resulting in an increased current level. The higher grading density within the resolution of screen-printing (~100 µm) was considered favorable to deliver higher power.

<u>2. Ink formulation Optimization of the TEGs</u>

The PTFE composite ink was optimized by varying the ratio of PTFE powder and the fluoropolymer in the formulation. In general, PTFE and other perfluorinated alkanes are considered among the most attractive negative electrode material due to its superior electron affinity. To endow printability, durability, and flexibility to the wearable module, a fluoroelastomer terpolymer that is comprised of vinylidene fluoride, tetrafluoroethylene and hexafluoropropylene was selected. Various ratios of the PTFE and the binder were tested under the same condition, as shown in **Supplementary Fig. 4**. It can be observed that the output peak voltage and peak current increase linearly with the weight ratio of PTFE, and maximized at 65 wt% with the peak voltage of 160 V and the peak current of 130 µA. The performance then decreased as more PTFE was added. This was caused by the deteriorated durability of the composite due to the lack of binder, where the printed composite peels off from the surface easily after undergoing repeated sliding. The formulation was hence optimized at 65 wt% of PTFE.

The EC-PU ink formulation was optimized by varying the ratio between the positively charged ethylcellulose (EC) powder and the positively charged polyurethane (PU) elastomeric binder, as shown in **Supplementary Fig. 5**. Similarly, as the weight ratio of the EC increased and PU decreased, the performance of the TEG module improved, reaching the maximum peak voltage of 185 V and peak current of 150 µA with 25 wt% of PU. This behavior is within expectation as

the EC has shown higher positive charge affinity compared to PU in the triboelectric series[5]. In addition, the decrease in PU binder creates lower friction between the mover and the stator and smoothens the sliding motion between two surfaces, therefore considered more favorable. Yet, not reflected in the data, the flexibility of the module deteriorates significantly with less binder, rendering the high-loading formulations unsuitable for wearable applications. To balance the mechanical durability and the energy-harvesting performance of the TEG module, the formulation of PU at 50 wt% was selected.

3. Performance Characterization and Optimization of the TEGs

The performance of the TEG with optimized ink formulation was evaluated in-vitro systematically. To simulate the arm-swinging motion of the TEG modules, sliding frequencies ranging from 0.83 Hz to 3 Hz were applied to the TEG modules, and the V_{oc} and I_{SC} were plotted against each frequency as shown in **Supplementary Fig. 6**. It was observed that the peak voltage did not change significantly whereas the current increases linearly with the frequency. This is mainly attributed to the linearly shortened time when the same amount of charge was transferred.

The washability of the TEG was also tested with 20-min cold water washing. Upon drying, the performance of the TEG has shown no change in the output voltage compared to that observed before the washing (**Supplementary Fig. 21**).

Although many of the work reports on the performance TEG devices in terms of their peak power, such information is not helpful to reflect the realistic performance of the module as an energy harvester. To fully gauge the performance of the TEG module in the microgrid system, its average output within a period of time was considered more useful for gauging its performance. The output of the TEG module was hence rectified with a bridge rectifying to inverse the pulses in the negative voltage region, and the rectified output was used to charge capacitors with different capacities. A 1.5 Hz, 60 s sliding session was implemented and the voltage change in the capacitors was monitored, as shown in **Fig. 2e**. It can be observed that the charging curves of capacitors were near-linear, indicating a constant current charging behavior from the TEGs. This phenomenon is attributed to the charge-generation mechanism of the TEG module, where a constant amount of charge was generated when the mover slide across the same area of the stator. To extrapolate the

applicable performance data from the charging curves, the total harvested energy within the period and the harvested energy were calculated with the equation:

$$E = \frac{1}{2}CV^2 \quad (1)$$

where *E* is the stored energy in the capacitor in J, *C* is a capacitance of the capacitor in F, and *V* is the voltage of the charged capacitor in V; and the average current was calculated with the equation:

$$I = C\frac{dV}{dt} \quad (2)$$

where *I* is the average current from the TEG module in A, *C* is a capacitance of the capacitor in F, and $\frac{dV}{dt}$ is the rate of voltage change in V s^{-1}. Using the above equations, the total amount of stored energy, and the average current was calculated and summarized in **Fig. 2f** in the main text. The average current from the TEG module did not change with capacitance, yet the amount of energy harvested within 60 s reached the maximum with the capacitance of 100 μF, indicating that the optimum pairing is near 100 μF for the TEG module.

Similarly, the performance of the TEG module with the optimized capacitance pairing was evaluated against various sliding frequencies using the same principles. A 100 μF capacitor was connected to the rectified TEG module and its charging curve was recorded, as shown in **Fig. 2g**. The stored energy and average current were calculated with **Eq. 1** and **2** and summarized in **Fig 2h** in the main text. The average current and the stored energy were observed to increase with the sliding frequency, which corroborated with the conclusion derived from **Supplementary Fig. 6**.

It is worth noting that as the TEG module behaves as a constant current energy source to the microgrid, its performance cannot be directly translated to power. For a TEG module powering a 100 μF capacitor at the frequency of 1.5 Hz, an average current of ~6 μA was obtained, which can translate to an average power of 6 μW when the capacitor is at 1 V, or up to 30 μW when the capacitor is charged 5 V. More in-vitro and on-body testing was performed in **Supplementary Note 4** and **5** to reflect the performance of the TEG module as a wearable energy harvester.

Supplementary Note 2: Fabrication, Characterization, and Optimization of the Biofuel Cells (BFCs)

1. Design and Fabrication of BFCs

The fabrication of the CNT pellets was adapted from previous work with minor adjustments[6]. The pellets were chosen for their 3-dimensional, high specific surface area property, which allowed a higher rate of reaction compared to a printed, planar design. Two types of CNT pastes were made as described in the main manuscript. As shown in **Supplementary Fig. 7**, a 1.67 mm thick PTFE sheet (McMaster Carr, Elmhurst, IL) with multiple punctured circular 5 mm-diameter holes was used as the stencil. PTFE was chosen as the stencil material due to its hydrophobicity, allowing the deposited paste not to stick to the wall of the stencil. The prepared pastes were printed through the PTFE stencil onto another sheet of PTFE substrate and were dried in the oven at 80 °C for 20 minutes. After drying, the pellets hardened and delaminated from the PTFE substrate. The anode pellets thus shrink to the diameter of 4 mm and thickness of 1.4 mm, while cathode pellets shrink to the diameter of 3.5 mm to a thickness of 1.0 mm. The average weight of anode and cathode pellets was measured to be 8 mg and 6 mg, respectively.

The fabrication of the current collectors of the biofuel cell requires the use of the SEBS resin, the flexible silver ink, and the flexible carbon ink. The formulation of the inks and the sequence of printing was described in the main manuscript and illustrated in **Supplementary Fig. 8**. Firstly, a layer of SEBS was cast onto the textile to smoothen the surface and avoid short-circuiting of the anode and cathode due to sweat-wetted textiles. The silver interconnection was then printed onto the SEBS layer, followed by printing the flexible carbon current collectors for the pellets to bond. Another layer of SEBS was printed on top of the exposed silver interconnections to fully insulate the silver lines which prevented them from interfering with the electrochemical reaction and protect them from the corrosion of sweat. A thin layer of carbon paste was then printed onto the flexible carbon as the bonding agent between the pellets and the carbon current collector. The anode pellets and the cathode pellets were then placed onto the uncured carbon paste, and the device was cured in the oven at 80 °C for 10 minutes to complete the assembly process.

Bovine serum albumin (BSA) solution was prepared by using 0.1 M potassium phosphate buffer solution (PBS) (pH = 7.4) and dissolve the BSA to a 20 mg mL^{-1} concentration. The lactate

oxidase (LOx) solution and the bilirubin oxidase solution were prepared in with the BSA solution in a 40 mg enzyme mL^{-1} BSA solution concentration. The glutaraldehyde (GA) solution and the Nafion solution were prepared by diluting them in ethanol to reach a concentration of 1 %. The chitosan solution was prepared by dissolving 1 wt% chitosan in 0.1 M acetic acid. The protoporphyrin IX (PPIX) solution was prepared by first mixing ethanol and acetone in a 9: 1 ratio by volume and dissolving the PPIX powder to a concentration of 40 mM. The artificial sweat were prepared in dissolving 85 mM NaCl, 13 mM KCl, 16 mM urea, and the desired concentration of lactate, into the 0.1 M PBS solution. All prepared solutions are stored in the freezer at -20 °C and thawed right before use. The solutions were drop-cast onto corresponding pellets sequentially as described in the main manuscript, with 5 minutes interval between each step. The drop-cast samples were stored in the refrigerator at 4 °C for at least 12 hours before use. The fabrication of BFC modules was then completed. The as-fabricated BFC module was shown in **Supplementary Fig. 9**.

2. Characterization and Optimization of BFCs

All in-vitro characterization of the BFC modules was performed using 0.5 M PBS (pH = 7.4) and were spiked with lactate solution to reach the desired concentration unless otherwise noted. A concentrated 0.1M lactate solution was prepared by dissolving concentrated lactic acid into the 0.5 M PBS. All lactate solutions in lower concentrations were prepared by spiking the 0.5 M PBS with the corresponding amount of the 100 mM lactate solution in PBS. When a 3-electrode electrochemical characterization was performed, using commercial silver/silver chloride (Ag/AgCl) reference electrode with 1 M of potassium chloride environment as the reference electrode, and a platinum wire was used as the counter electrode. When 2-electrode characterization is performed, the BFC cathode was used as the working electrode while the BFC anode served as the counter and reference electrode.

The BFC module was designed to have an anode to cathode ratio of 1: 4. This was designed to compensate for the rate-limiting oxygen reduction reaction (ORR) reaction taking place on the cathode. This limited reaction rate can be observed by characterizing the anode pellet and the cathode pellet individually with linear sweep voltammetry with a scan rate of 5 mV s^{-1}. The anode pellet was scanned from -0.3 V to 0.3 V and the cathode pellet was scanned from 0.6 V to 0 V vs.

Ag/AgCl both in a PBS environment and 20 mM lactate environment. As shown in **Supplementary Fig. 10**, the current response of the cathode is noticeably lower compared to the anode.

The performance of each BFC module was then characterized by both LSV and chronoamperometry (CA) in a 2-electrode system. Traditionally, most of the wearable BFC devices are characterized by LSV, with various scan rates ranging from 5 mV s^{-1} to 0.2 mV s^{-1}.[6–8] However, for high-surface-area electrodes like the CNT pellets used in this work, this method will result in a large discrepancy based on the scan rate selected. The power of the BFC was calculated using the formula.

$$P = V \times I \quad (3)$$

Where P is the power in W, V is the applied potential in V and I is the current in A.

As shown in **Supplementary Fig. 11**, as the scan rate changes, the obtained power at a scan rate of 5 mV s^{-1} is more than 25 times higher than that obtained from 0.2 mV s^{-1}. If characterized with LSV only, the reported power would be 1.41 mW cm^{-2} based on the cathode area or 3.18 mW/cm^{-2} based on the anode area. The majority of this power was contributed by the capacitive current from double-layer capacitance due to the high surface area of the 3D CNT pellets and does not necessarily reflect the true performance of the BFC module when being discharged continuously. To avoid this problem, after scanning at the lower speed to locate the range of the voltage of the maximum power (~ 0.55V), we performed chronoamperometry set at different voltages ranging from 0.25 V to 0.64 V with increments of 0.05V. The resulting CA plots are shown in **Fig. 3d**. We noticed that the power output of the BFC module took > 3 minutes to stabilize, where the majority of the capacitive currents were consumed, and the redox currents became dominant. The power at the endpoints of each 10-minute scans was used as the true power of the BFC module at the set voltage and was summarized to form **Fig. 3e** in the main text. The power of the BFC module at 15 mM lactate concentration demonstrated the power of 21.5 μW per module discharged at the voltage of 0.5 V, which has a ~35 times difference compared to the 5 mV s^{-1} LSV scan. With this observation, we would like to state that although using CA as a characterization method would be more realistic in reflecting BFC's power when discharging continuously over a long period of time, especially for electrodes with high surface areas. This practice can be more time consuming and disadvantageous in reporting the maximum power yet should be more

advocated as it accurately reflects the performance of BFCs as energy harvesters. The accurate measurement of the power of the BFC system is of great importance when integrating with other energy harvesters to ensure commensurate power rating compared to other energy harvesters.

The voltage of discharging the BFC modules is of high importance for maximizing the efficiency of the BFC modules when integrating with energy storage devices. As shown in **Supplementary Fig. 13**, the BFC showed the less response to the addition of lactate fuel compared to at 0.4 V and 0.6 V. This also corroborated with the data obtained from **Fig 3e**, where the highest current response is found near 0.5 V. The endpoints of each scan in **Fig 3f** in the main text is selected to form **Supplementary Fig. 12**. In order to use a voltage boosting circuit with the BFC module with maximized efficiency, this optimization is crucial as a minimum cut-off voltage is to be set to the voltage boosting circuit to avoid over-discharging the BFC module at a lower potential. More of the circuit settings for the BFC module is discussed later in **Supplemental Note 4**. Overall, under optimized discharge voltage, the power of the module increases with the lactate concentration, from 9.7 μW at 5 mM to 25.3 μW at 25 mM. Generally, the realistic lactate concentration can vary between 2-3 mM to up to 20-30 mM and are dependent on several factors based on the fitness of the human subjects, their gender, age, level of hydration, as well as the type of exercise, location of perspiration, and the time into the exercise. This phenomenon has been studied extensively in various studies, and hence is not discussed in detail here[9–11]. To simulate the performance of the BFC in real human sweat, an artificial sweat was formulated using 85 mM NaCl, 13 mM KCl, and 16 mM urea, with 0 mM and 20 mM lactate. As shown in **Supplementary Fig. 15**, the BFC demonstrated similar performance in the artificial sweat as in the PBS, indicating that the other components other than lactate in of the sweat have insignificant impact to the performance of the BFC.

The stability of the BFC was also tested using CA throughout a week. The BFC under test was stored in a refrigerator under 4 °C and was taken out for testing every 24 hours in a 10 mM lactate environment. The results of the individual CA measurements are summarized in **Supplementary Fig. 14**. The washability of the BFC was also tested in 20-min of cold water washing without the addition of detergent. The performance of the BFC before and after washing at 0 mM and 20 mM lactate in 0.5 M PBS was measured via CA, and the results have shown no change in the output power after the washing (**Supplementary Fig. 21**).

Supplementary Note 3: Fabrication and Characterization of the Supercapacitors (SCs)

1. Design and Fabrication of SCs

The electrode formulation and fabrication of the supercapacitors (SCs) was adapted from previous reports with minor adjustments[12,13]. Multiwalled carbon nanotubes (CNT) were chosen for its high surface area and good conductivity as an electrode material. The carboxylate-functionalized variation of the CNT was chosen for its better dispersity in aqueous solvents. A screen-printable poly(3,4-ethylene dioxythiophene) polystyrene sulfonate (PEDOT:PSS) gel is chosen as the binder which offers flexibility, pseudocapacitive behavior and high conductivity. A fluorosurfactant (Capstone FS-65) was chosen as it promotes the dispersion of CNT in the PEDOT:PSS gel as well as the phase separation between PEDOT and PSS which results in superior stretchability and conductivity[14,15]. The ink was formulated layer-by-layer as described in the main text. Firstly, a layer of SEBS is cast onto the textile to smoothen the surface and avoid sweat short circuiting, similar to the BFC modules. The electrode ink, flexible silver ink, and electrolyte gel were then printed onto the substrate and cured in their corresponding temperatures as described in the main text. As the electrolyte dries in air, the capacity of the SC gradually increases and stabilize as it solidifies, which takes 8~12 hours under ambient environment[16]. Oven-drying at elevated temperature is not recommended as it may cause the PVA gel to degrade and change color. After drying, the electrolyte became flexible and stretchable. After the electrolyte solidifies, another layer of SEBS was cast onto the printed SC module to enhance its flexibility by enhancing the bonding between electrodes, electrolyte and the substrate, and to protect the SC module by prevent any sweat-induced degradation or dissolution when used on-body. The fabricated module is composed of 5 SC units connected in series, and each module can be connected in parallel or in series to adjust its capacity. The fabrication process and possible configurations of the SC modules are shown in **Supplementary Fig. 16**, and corresponding photo images in **Supplementary Fig. 17**. The fabricated SC samples are thereafter ready for characterization.

2. Characterization of SCs

Two testing methods: cyclic voltammetry (CV) and galvanostatic charge-discharge (GCD), have been employed onto one SC unit for characterizing the areal capacitance of the printed CNT-PEDOT:PSS electrodes. CV with the scan rates of 5, 10, 25, 50, 100 mV s^{-1} between the window of 0 V and 1 V were carried out. The areal capacitance is calculated using the formula:

$$C = \frac{1}{2\nu A(E_f - E_i)} \int_{E_i}^{E_f} I \, dV \quad (4)$$

where C is the areal capacitance in F cm^{-2}, v is the scan rate in V s^{-1}, A is the area of electrodes in cm^2, E_f is the higher vertex potential in V, E_i is the lower vertex potential in V, and the I is the current in A. To confirm the areal capacitance, GCD was conducted at charge/discharge between 0 V and 1 V with currents at 25, 50, 100, 250 and 500 μA. The capacitance at discharge is used to gauge the capacitance of the SC unit, which is calculated using the equation:

$$C = \frac{I\Delta t}{A(E_f - E_i)} \quad (5)$$

where C is the areal capacitance in F cm^{-2}, I is the current in A, Δt is the time taken for the discharge, A is the area of electrodes, E_f is the charged potential in V, and E_i is the discharged potential in V. The resulting areal capacitance measured by both methods are summarized in **Supplementary Fig. 18** of the main text. The area of each SC unit is designed to be 0.375 cm^2, which translates to an areal capacitance of ca. 10 mF cm^{-2} for the printed CNT-PEDOT:PSS electrodes.

The performance of the whole SC module which composed of 5 SC units was also tested with GCD, as shown in **Supplementary Fig. 19**. The capacity of the SC module is calculated using **Eq. 5**, and the coulombic efficiency (CE) of the charge-discharge cycle is calculated using the equation:

$$CE = \frac{t_{discharge}}{t_{charge}} \times 100\% \quad (6)$$

where $t_{discharge}$ is the time take to discharge from maximum voltage to 0 V in s, and t_{charge} is time to charge from 0 V to the maximum voltage in s. The capacity of the SC module remained near 150 μF at different maximum voltage, with the CE gradually decreased as with the maximum voltage down to near 91 % at the voltage of 5 V.

The washability of the SC was also tested in a 20-min cold water washing test. The performance of the SC before and after washing was measured using GCD, and the results have shown no change in the capacitance after the washing (**Supplementary Fig. 21**).

The self-discharge of the module is tested, as shown in **Supplementary Fig. 20**. The module is firstly charged to 5 V with a 25 μA current, and then left discharge without an external load with the potential of the module monitored using the potentiostat. The capacitor lost 50% of its charge within 4 hours, mostly due to the faradaic process took place in the electrolyte in the form of diffusion[17]. This rate of self-discharge can vary based on the rate of charging, the state of the SC before the discharge, *etc.*, and can be studied in further details[18]. Here, the rate of potential change and the corresponding self-discharge current is calculated with the equation:

$$I_{SD} = C\frac{dE_{SC}}{dt} \tag{7}$$

Where I_{SD} is the equivalent current for self-discharge in A, C is the capacitance of the tested module in F, E_{SC} is the potential of the module in V, and t is the time in s, and their relationship to the state of charge is presented in **Supplementary Fig. 20b**. As seen in the figure, the self-discharge rate increases exponentially with the supercapacitor voltage, with the equivalent self-discharge current drops to nearly an order of magnitude as the potential of the SC module drop to half. The rapid self-discharge is a common behavior shared by capacitors and supercapacitors, which limits their ability to serve as a long-term energy storage unit. However, in the wearable microgrid system described in this work, the SC module was dominantly used for regulating the energy scavenged by the harvesters, thus the long-term energy storage behavior is deemed less crucial to the operation of the system.

It is worth noting that although the performance of the SC modules may not be the most state-of-art compared to other reports, the main focus of this work is the commensurate, complementary, and synergistic integration of all components. The fabricated SC modules were qualified for meeting this requirement in terms of wearable, flexible form factors with the appropriate amount of capacity and voltage for integration, hence were not further optimized. More advanced modifications can be applied to enhance the flexibility, rechargeability, or the areal capacitance of the SC modules, and more exhaustive, in-depth characterization can be applied to fully understand the performance of the module.

Supplementary Note 4: Characterization and Discussions of the In-Vitro and On-body Operations of the Integrated Bioenergy Harvesting-Storage Modules

1. In-vitro Testing Setup

The performance of integrated modules was characterized both in-vitro and on-body in order to find the optimum pairing of the modules and to demonstrate the complementary, commensurate and synergistic behavior of the system. Similar to previous sections, the TEG module was regulated by a bridge rectifier, and the rectified output was directly connected to the SC module for charging. As most of the commercial electronics requires a higher voltage (1.5 - 5 V) than the potential of any BFC can provide, the BFC module requires a voltage boosting circuitry to match the desired high voltage for charging the SC. Based on previous studies, a low-power boost charger chip with battery management function, bq25505 from Texas Instruments, was chosen for regulating the output of the BFC module[6,8]. The circuit diagram of the booster is illustrated in **Supplementary Fig. 22**. It is worth noting that the efficiency of the booster circuit varies with the input voltage, input current, as well as potential of the storage module. Generally, for a BFC module with 0.5 V input voltage and 30 μA input current, the efficiency varies between 50% - 80% based on the potential of the SC module[19]. By using the battery management function included in the chip, a maximum potential of 5.1 V was set to avoid overcharging the SC module. A reference voltage of 0.5 V was set for the power input, hence regulating the circuit to discharge the BFC module at a constant voltage to ensure maximum input power from the BFC, as discussed in previous sections. The connection of the modules is illustrated in **Supplementary Fig. 23**, where the anode of the BFC, SCs, and the negative ends of the rectifier were connected to the ground of the circuit, and the positive output from the booster and the rectifier were connected to the cathode of the SC.

The in-vitro testing of the system was performed on the desktop, where the frequency-controlled sliding motion was applied to the mover of the TEG on top of the stator to simulate arm movements, and PBS solution spiked by lactate was used to simulate human perspiration BFC module. Two TEG modules and one BFC module were connected to the microgrid as energy inputs, and one SC module was connected as the energy storage module. The potential of SC was monitored during the in-vitro simulation session to study the behavior of the system during different

scenarios. The sliding frequency of 1.5 Hz was applied onto the TEG module to simulate the speed of arm swinging during a running exercise. A lactate concentration of 15 mM was selected to simulate the lactate concentration in human sweat. For each BFC module, 1 mL of 0.5 M PBS was applied to cover the electrode surface, and the corresponding amount of 100 mM lactate solution was spiked to the PBS to reach the target lactate concentration. It is worth noting that when the SC voltage is below 1.8 V, the booster requires a cold-start phase, where the energy from the BFC is used to jump-start the system. This process depends on the left-over charge in the system capacitor, input- from the BFC module, and other factors, which cannot be precisely controlled. For the clarity of the data, the in-vitro tests were performed by pre-charging the capacitor to 1.8 V to avoid the cold-start phase.

2. Additional Discussions of the In-vitro System Performance

As shown in **Fig. 5a**, three scenarios were proposed to simulate the starting, during, and ending of a movement session. The additive effect of combining two types of energy harvesters can be extrapolated from the presented data, where the charging rate of the SC module is noticeably faster compared to having only the TEG or the BFC bioenergy harvesters. Yet, more importantly, the data is illustrative of the synergistic effect of combining two harvesters that are complementary in harvesting bioenergy from human movements. The characteristic advantage and limitation of the motion-induced triboelectric effect were clearly shown in the scenario (i) and (iii), where the energy supply to the SC module starts and stops immediately upon starting and stopping of the movements. The BFC module itself, although not limited in the activation speed, was more constrained by the availability of the lactate in realistic situations, as the increase in lactate concentration and sweat rate lags behind the start of movements.

The complementary behavior of the harvesters was shown across 3 different settings with different lactate concentrations and sliding frequencies. For a system designed to charge quickly with the input of energy, the TEGs can compensate for the delayed booting of the BFC module, which was stimulated by the delayed spiking of lactate into the PBS in scenario (i), and allowing the system to reach a higher state of charge within the time frame of the simulation. On the other hand, when the movements stop or temporarily pause, the stable power output from the BFC module is able to compensate for the TEG modules to continuously provide power to the SC

module, as simulated by scenario (iii). Such behavior is desirable for system operating with continuous discharge (**Fig. 6e**), and is extremely crucial for high-power applications where the SC serves as a short-term energy reservoir for pulsed high-current discharge. A system reacts and spontaneously with movements was not able to sustain repeated discharge after movement stops (**Fig. 6f**).

3. On-body Testing Setup

Polyvinyl alcohol (PVA) hydrogel was fabricated based on previous work for the on-body operation of the BFC modules[8]. A 10 wt% PVA (MW 89,000 – 98,000) was prepared by dissolving in hot water and was mixed with an equal amount of 20 wt% potassium hydroxide (KOH) solution by weight to form the hydrogel precursor. The precursor was dried in a desiccator under vacuum to cross-link into the gel, and the gel was soaked in deionized (DI) water several times to remove excess KOH, and then soaked in 0.5 M PBS for later use. The hydrogel can serve as a stable electrolyte to maintain the ionic connection between the anode and the cathode pellets, and also as a reservoir to absorb and temporarily store the sweat from the human body.

The printed BFC, TEG, and SC modules were attached to a stretchy, nylon-based shirt by adhesives, with their corresponding position shown in **Fig. 1c**. Printed, stretchable silver traces and enameled copper wire (36 AWG) was used to connect individual components in the same configuration as the in-vitro settings, and the printed traces and the contact points of the enameled wires were further insulated with SEBS to avoid short-circuiting from sweat. The TEGs were aligned so the natural arm-swinging motion is mostly perpendicular to the interdigitated patterns on the TEG stator so that the patterns on the TEG movers are parallel to the patterns on the stators when in contact. A cycling exercise machine is used to quickly induce sweat in a movement session, and the test subject is asked to ride the cycling machine while swinging their arm along with a metronome set at 180 beats per minute (one stride per beat) for a designed length of time.

Two leads were extended from the SC electrodes and connected to a potentiostat for pre-charging/discharging the SC module (if needed) and monitoring its voltage during the charging period. As shown in **Fig. 5b**, data was generated by pre-charging the module to 2 V before the movement starts. This was done for demonstrating better the synergistic and complementary effect of the system by avoiding the cold-start phase of the booster circuit, similar to the in-vitro tests

described above. In the normal use-case, the SC modules were to be charged from 0 V instead of 2 V in a realistic situation, which was tested, and the results are presented and discussed in the following section.

4. Additional Discussions of the On-body System Performance

In **Fig. 5d**, it is worth noting that the erratic cold-start behavior of the booster was pronounced when starting from 0 V, with most of the BFC-charged SC stuck in the 1.8 V cold-start mode until sufficient current can be supplied from the BFC modules due to the increase in lactate concentration from the exercise. The timing of the booster exiting the cold-start phase can vary based on the perspiration time and rate, and upon the lactate concentration of the test participant; these are variables difficult to be regulated, as they depend on the physiological state of the participants at that moment. The performance in the TEG module was also observed to have declined compared to the in-vitro testing, reflecting the change in pressure and less aligned movement between the stator and the mover.

In general, the charging time of the SC modules increases with the capacitance. For the system to reach operation voltage within a reasonable amount of time, it is best advised to use the SC module with capacitance as low as possible to ensure the fast booting endowed by the TEG modules to be fully exploited. Hence, the power consumption of the application used in the system is to be accurately measured and the energy carefully budgeted to design the microgrid with a necessary but minimum amount of capacitance, which allows the system to operate smoothly from the start to the end of an activity session.

Supplementary Note 5: Design, Fabrication, and Characterization of The Wearable Applications in the Microgrid

1. Design, Fabrication, and Characterization of the sodium ion (Na^+) sensor

The fabrication of the sodium ion sensor is illustrated in **Supplementary Fig. 24**, which was adapted from previous works[20,21]. The design of the Na^+ sensor was made into a 150 μm-thick metal stencil for screen-printing. Firstly, a layer of the SEBS resin was cast onto the textile substrate using an adjustable doctor blade with a thickness of 200 μm and cured in an oven at 80 °C for 10 min. Then, the collector and reference electrode were printed using the flexible silver ink cured at 60 °C for 20 min. The flexible carbon composite ink was printed as the working electrode and was cured at 80 °C for 20 min. Another layer of the SEBS resin was printed onto the sensor to insulate the interconnections and the junction between the carbon and the silver layer and cured at 80 °C for 10 min.

To functionalize the reference electrodes, the exposed region of the flexible silver electrode is partially reacted with iron (III) chloride ($FeCl_3$) to form a silver/silver chloride electrode. 5 μL mm^{-2} of 0.1 M $FeCl_3$ solution was drop cast onto the electrode and let react for 1 min before rinsing off with DI water. A cocktail solution was prepared which was consisted of 78.1 mg polyvinyl butyral (PVB) and 50 mg sodium chloride (NaCl) dissolved in 1 mL of methanol. The NaCl was added in excess to ensure saturation. 1.5 μL mm^{-2} of the cocktail was then drop-cast onto the Ag/AgCl surface. 0.5 μL mm^{-2} of the water-based polyurethane was then drop-cast onto the electrode after the cocktail is dried. Another cocktail for the ion-selective working electrode was prepared by dissolving 1 mg sodium ionophore X, 0.55 mg sodium tetrakis[3,5-bis(trifluoromethyl)phenyl]borate (Na-TFPB), 33 mg polyvinyl chloride (PVC) and 65.45 mg dioctyl sebacate (DOS) dissolved in 660 mL of nitrogen-purged tetrahydrofuran (THF). 2.5 μL mm^{-2} of such cocktail was drop cast onto the carbon working electrode. The decorated completely evaporate the solvents (e.g., THF and distilled water) at room temperature before use.

The response of the Na^+ sensor was tested by measuring the open-circuit potential (OCP) between the working and reference electrodes after being exposed to solutions with various Na^+ concentrations as shown in **Supplementary Fig. 25**. The membrane on the working electrode can selectively exchange Na^+ with the external aqueous solution by the use of ion-selective sodium ionophore and the Na-TFPB ion-exchanger dissolved in PVB-DOS in the organic phase. The potential of the sensor is hence dependent on the concentration gradient between the aqueous phase and the organic phase, described by the Nernst equation:

$$\Delta E = \frac{RT}{zF} ln \frac{[Na^+_{(aq)}]}{[Na^+_{(org)}]} \tag{8}$$

Where ΔE is the change in potential in V, R is the gas constant = 8.3145 J mol^{-1} K^{-1}, T is the temperature in K, z is the charge of the ion, F is the Faraday constant = 96485 mol^{-1}, $Na^+_{(aq)}$ is the sodium concentration in the tested solution, and $Na^+_{(org)}$ is the sodium concentration in the ion-selective membrane, which should be constant[22]. The potential of the sensor hence changes logarithmically with the Na^+ concentration, with a Nernstian response of 0.05913 V per decade at 298 K in the ideal situation.

Solutions of NaCl with the concentrations of 0.1 mM, 1 mM, 10 mM and 100 mM were prepared by dissolving NaCl in DI water. The sensor is firstly conditioned with 100 mM NaCl solutions for 30 minutes to saturate the Na^+ in the ion-selective membrane and rinsed with DI water. The sensor was then tested in different concentrations with increasing and then decreasing NaCl concentration, as illustrated in **Supplementary Fig. 25c**. The response of the sensor is recorded to form the calibration curve (**Fig. 6d**) in the main text, which is shown to have a linear response of 0.05719 mV per decade of concentration change by using the least-squares fitting with an R^2 score of 0.99716. The reversibility of the sensor was also tested by having the sensor repeatedly exposing the sensor to 0.1 mM, 1 mM, and 10 mM NaCl solutions. As shown in **Supplementary Fig. 25d**, this resulted in a negligible hysteresis to such changes in concentrations.

2. Design, Fabrication, and Characterization of the Electrochromic Display (ECD)

As a simple demonstration of a complete wearable, self-powered application, a flexible sensor-display system was developed that reports the signal of the sensor directly via a low-energy consumption display. An all-printed PEDOT:PSS-based ECD system was developed based on

previous reports[23,24]. A transparent thermoplastic polyurethane sheet was selected as the substrate. The ECD electrode ink was formulated by mixing 0.5 wt% of Capstone fluorosurfactant into screen-printable PEDOT:PSS in the mixer for 5 min at 2500 rotations per minute (RPM), and sieved through a syringe filter with 1 μm pore size. The ECD electrolyte ink was formulated by mixing poly(sodium 4-styrene sulfonate), water, D-sorbitol, glycerol, and titanium dioxide (TiO_2) in 3: 2: 1: 1: 0.35 ratio by weight with the mixer for 5 min at 2500 RPM. A white opaque insulator ink was formulated by mixing 5 wt% of TiO_2 into the SEBS resin.

The inks were printed layer-by-layer onto the PU substrate using a designed 100 μm-thick metal stencil, as illustrated in **Supplementary Fig. 26**. The ECD is composed of the front electrode panel and the back panel with 8 individual electrodes to assemble into 8 electrochromic pixels. To fabricate the back panel, 8 PEDOT:PSS electrodes were printed with the ECD electrode ink and cured in the oven at 80 °C for 60 min. Flexible silver ink as connections and the opaque insulation ink was then printed consecutively and cured at 80 °C for 10 min each. The top panel was printed with the ECD electrode ink and the flexible silver ink with the same curing condition as above. A layer of the ECD electrolyte ink was printed onto the back-panel pixels and two panels were carefully aligned and attached. A heat sealer was used to seal the ECD on all 4 sides, allowing the ECD to be flexible without dislocation between two electrodes.

The operation of the ECD pixels follows the simple reversible redox reaction of the PEDOT:PSS,

$$PEDOT^{+}PSS^{-} + Na^{+} + e^{-} \leftrightarrow PEDOT^{0} + Na^{+}PSS^{-} \tag{9}$$

where the oxidation reaction takes place when the positive signal is applied to the positive electrodes on the back panel, and the reduction reaction takes place at the corresponding location of the electrode on the front panel and turns its color from light blue to dark blue.

The electrochemical performance of the ECD pixels was tested via CV and CA, as shown in **Supplementary Fig. 28**. A 2-electrode CV scan between 0 and 2V is performed to the system, showing the oxidation of the electrode with a broad, non-specific oxidation peak near 1 V, and a sharper reduction peak at 1.2 V. The turn-on and turn-off response was monitored by high-speed CA with a different applied voltage of 1 V, 1.5 V, and 2 V to “turn-on” the pixel and 0 V to “turn-off” the pixel. As shown, when the potential is applied, the reaction set off rapidly with the majority

of the reaction took place within the first 0.1 s and the color of the pixel changes to dark blue instantly. After 0 V is applied, the dark blue color changes back to the original translucent blue color quickly, with the majority of the reaction completed within the first 0.2 s. As demonstrated in **Supplementary Fig. 28c**, the amount of charge transfer increases with the applied potential, indicating a higher amount of power consumption and longer turn-off time when applying a higher potential. As the majority of the reaction takes place within the first 0.1 s, a transient application of on/off signal is sufficient to refresh the status of the ECD, allowing the system to be powered in a quick, pulsed discharge of a capacitor.

3. Power Consumption of Wearable Applications and Microgrid Energy Budgeting

The measurement of power consumption is crucial for the design of the microgrid and the selection of appropriate components for integration. Two applications that represent two different use case of the microgrid were selected: a wristwatch that requires constant low-power supply, and the developed wearable integrated sensor-ECD system that requires pulsed refresh sessions with instantaneous higher power consumption. The minimum amount of energy is to be calculated and the appropriate SC module to be pair to ensure both successful operation and fast booting.

The power consumption of the wristwatch was measured by removing the battery and connecting the watch to the potentiostat. Different voltages were applied to the watch and their corresponding current consumption was measured using CA, as shown in **Supplementary Fig. 29**. The circuit in the watch discharged in a pulsed fashion, with the average power consumption varying from 10 μW at 4 V to 1 μW at 2 V. As shown in **Fig. 6b**, the liquid-crystal display (LCD) of the watch was not able to display with proper contrast below 2.5 V. The lithium metal primary battery used for the watch is rated with a nominal voltage of 3 V, which corresponds to the average power consumption of ca. 3 μW. Different capacitors ranging from 10 μF to 220 μF charged to 3 V were connected to the watch and their voltages recorded, showing that even a capacitance of 47 μF was able to maintain its potential to nearly a minute. For this reason, the configuration of the SC modules with the amount of capacitance of 75 μF was selected for buffering and regulate the input energy from the TEG and the BFC harvesters.

The power consumption of the pulsed discharge mode application was to be characterized more precisely, as it has a higher requirement in the amount of energy in one operation session. A

low-power microcontroller AtTiny441 from Atmel was selected for the sensor-ECD application. The microcontroller features built-in 4 bytes programmable flash memory for a pre-uploaded program which can effectively translate the analog output from the sensor to the digital output to the ECD pixels, as well as a 10-bit analog-to-digital converter that allows mV signal resolution required to differentiate the signal from the sensor with different Na^+ concentrations. The set-up of the microcontroller is illustrated in **Supplementary Fig. 28**. The controller was pre-programmed to display potential up to 0.32 V, with each ECD pixel corresponding to one 0.04 V increment of the sensor output.

The power consumption of the microcontroller connected to the sensor-ECD system was measured similarly using the potentiostat set at different voltage as demonstrated in **Supplementary Fig. 31**. As shown, the power consumption of the microcontroller exceeded the wristwatch by 3 orders of magnitude, ranging from 4 mW at 2 V to 30 mW at 5 V. Yet, the microcontroller was able to boot quickly within the first 50 ms, take readings and apply signals to the ECD pixels within the first 200 ms, which allow this system to be powered transiently from the discharge of one charged capacitor. The energy consumed by one discharge session can be estimated by the equation:

$$E = P \times t \tag{10}$$

where E is the energy in J, P is the power in W, and t is the time in s. To supply 200 ms of operation assuming the lowest power of ca. 4-5 mW, the energy required was thus estimated to be ca. 0.8 – 1 mJ.

Capacitors ranging from 100 μF to 470 μF were able to supply sufficient energy to the microcontroller in one continuous discharge session but the larger capacitors had shown no substantial extended operation time (**Supplementary Fig. 31b**). The capacitors were discharged with 200 ms pulsed discharge, where more differences were more pronounced. The higher capacitance capacitors were able to sustain multiple refresh sessions, and the number of sessions decreases with the capacitance, as to 1 successful discharge session from the 100 μF with a slight excess amount of energy, and 1 discharge from the 47 μF capacitor which did not last throughout the entire 200 ms session (**Supplementary Fig. 31c**). It is thus determined that the 75 μF SC modules should retain enough energy to sustain one complete refresh session.

The required state of charge of the SC was then characterized by charging the 75 μF module to different potentials, and use the SC to power one refresh session, while the color on the ECD recorded to determine the minimum state of charge required for the 75 μF SC module to induce color change with sufficient contrast (**Supplementary Fig. 32**). It can be observed that the contrast of the on and off pixels gradually decreased with the initial state of charge of the SC modules, with the difference barely recognizable below 3.5 V. The minimum potential of 4 V before initiating a refresh session was thus decided. To confirm the calculation above on the energy required for one operation session, the energy stored in the capacitor was calculated using the equation:

$$E_{SC} = \frac{1}{2}C(V_i^2 - V_f^2) \quad (11)$$

where E_{SC} is the energy discharged from the SC in J, C is the capacitance of the SC in F, V_i^2 is the potential of the SC before the discharge in V, and V_f^2 is the potential of the SC after the discharge in V. For a 75 μF SC to discharge from 4 V to 1.25 V, the energy released from the discharge is thus calculated to be 0.88 mJ, which agreed with the previous calculation based on **Eq. 10**.

4. On-body Testing Setup and Discussions

The complete wearable microgrid was assembled onto the shirt by sewing and connecting by enameled wires, similar to the on-body testing in **Supplementary Note 4**. The applications were connected to the microgrid as illustrated in **Supplementary Fig. 33**.

To power the wristwatch via the bioenergy microgrid, 7 min of cycling activity was carried out while the potential of the SC modules was monitored. The SC was charged from 0 V instead of 2 V to reflect the realistic booting scenario of the microgrid. The benefit of the complementary and synergistic effect between the TEG modules and the BFC module was evident when comparing to the single-harvester operation mode: in the beginning, the TEGs allowed the booster to quickly exit the cold-start phase and raise the potential to above 2.5 V where the LCD of the watch is operational; after movement stopped, the BFC was able to extend the operation time of the watch for at least 20 more minutes. With optimized capacitance paired with the system, the microgrid was able to boot quickly within 3 min, compared to the expected 5-10 min if configurations with larger capacitance were used.

Similarly, to power the sensor-ECD application, a 10 min cycling activity was carried out while the potential of the SC modules was monitored. The integrated harvesters allow the system to quickly boot in ~3 min to 4 V to supply for the first sensing-display refresh session. It was able to maintain ~10 refresh sessions within the 10 minutes of exercise, and > 10 sessions in the later 20 min with a slower refresh rate even after movement stopped. In comparison, the BFC harvester alone was not able to boot until 7 min later towards the end of the activity session, and the TEG harvester was able to boot quickly within 2.5 min but was not able to sustain more discharge sessions once after the movement stopped.

Although demonstrated with the combination of TEG and BFC to power the low-powered wearable applications, the implication of this work lies beyond this specific example. In this presented work, the system is designed to be operational during and after an activity session, which is independent from the external environment and optimal for sweat sensing for athletic applications, especially in an indoor environment where common environmental energy input is unavailable. It can be limited in other scenarios when movements or sweat are unavailable, but its operation will not be needed as the sweat sensor has no analyte and no movement to induce sweat electrolyte or metabolite change. Other integrations should be considered when designing the microgrid for other applications.

While the integration of various flexible or wearable energy harvesters has been proposed previously, most of these studies stopped at in-vitro level proof-of-concept, with a few more outstanding work successfully integrating the modules into a complete wearable device and has shown their operation on-body. However, such integrations usually lack discussion on the reasoning behind the selection of components and applications, and how this integration would be beneficial for their designed use case. Integration of wearable TEG with another motion-based energy harvester, as an example, is beneficial for harvesting an additional amount of energy, yet at a cost of occupying the area that can be used by simply expanding the TEG or the other harvester (whichever one with higher power). Such integration is novel and a great proof-of-concept, yet not realistic for they integrate merely for the sake of novel integration. This concern sometimes can be extended to systems that integrate harvesters that operate on different harvesting mechanisms (e.g. TEGs with solar cells, BFCs with thermoelectric generators). These systems can have more efficient energy scavenging as they can convert energy from different sources into electrical energy.

However, the specific use case for these integrations should be discussed, such as the integration of TEGs with solar cells should only be used for sunny, outdoor activities and the BFC with thermoelectric generators are only to be used in hot, sweaty conditions. Moreover, such integration should be paired with applications needed in such scenarios, such as a UV sensor for sunlight or a temperature or electrolyte sensor to avoid dehydration under high temperatures.

Aside from the selection of types of components, the energy rating or individual components are also crucial. For instance, it is most likely to be pointless to integrate a 10-μW BFC with a 100-mW solar cell, as the latter is overpowering enough to deem the former insignificant. More commonly seen situation is the pairing of harvesters with energy storage units with high capacity ratings, resulting in the booting speed going up to hours or the charging time up to days, which is unrealistic for wearable applications. The applications should also have commensurate energy and power rating, and situations of oversupply or undersupply such as to use an mW-level harvester to power nA-current sensors or to charge an Ah-level cell phone battery should be avoided. It would be equally absurd to use a gasoline generator to power a headphone jump start a car with a AAA battery. Future work should also consider employing more complex energy management systems, where multiple energy storage mechanisms are adapted for both high-power, short-term storage and high capacity, long-term storage.

To summarize, the notion and incentive to propose the concept of a wearable microgrid concept is to advocate beyond the novel form factors –considerations in the selection of components, as well as judicious budgeting of the scavenging and consumption of energy in the system, are just as important. Such design concepts are essential to push novel wearable devices towards realistic, market-ready wearable products, instead of letting it become the hype of yesterday. This implication can also be expanded to the study of an integrated portable system or implanted systems. Adapting the concept of microgrid, the future miniaturized systems will be one more step closer to a truly sustainable, independent, and energy-smart system.

Supplementary Figures

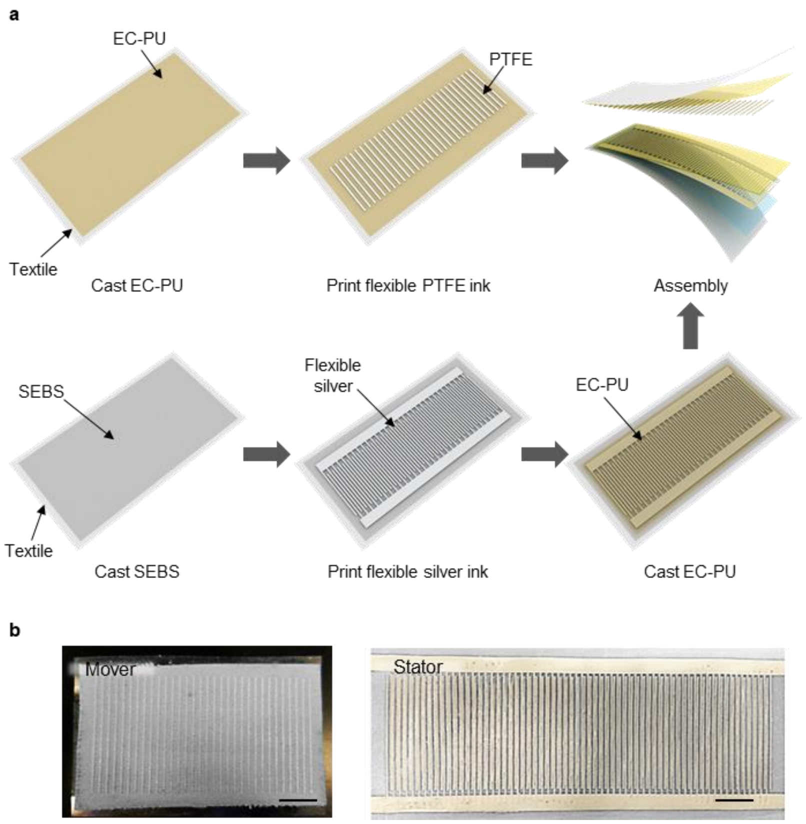

Supplementary Fig. 1 Screen-printing fabrication of the TEG module.(a) The layer-by-layer printing process of the mover (top) and the stator (bottom) of the TEG module. (b) Photo images of the printed mover and stator. Scale bar: 1 cm.

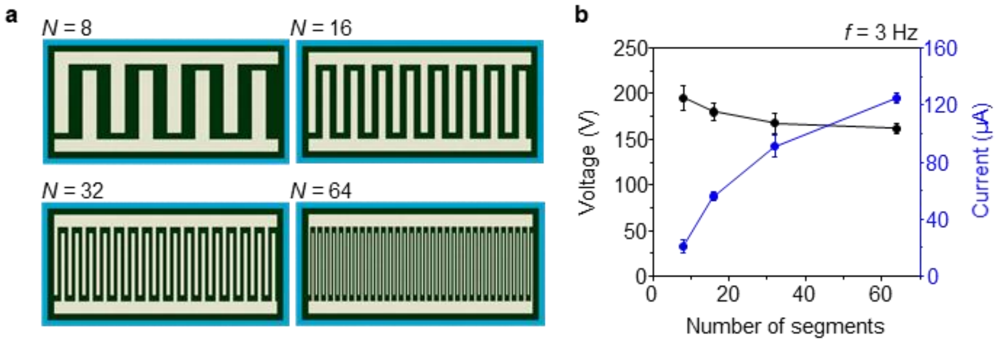

Supplementary Fig. 2 Structural optimization of the TEG module with different interdigitated grating units (*N*). (a) The schematic images of TEG stators with different number N of interdigitated lines within the length of 10 cm (*N* = 8, 16, 32, 64). (b) The measured peak V_{OC} and I_{SC} of TEG module with different line density. The operational frequency is fixed at 3 Hz.

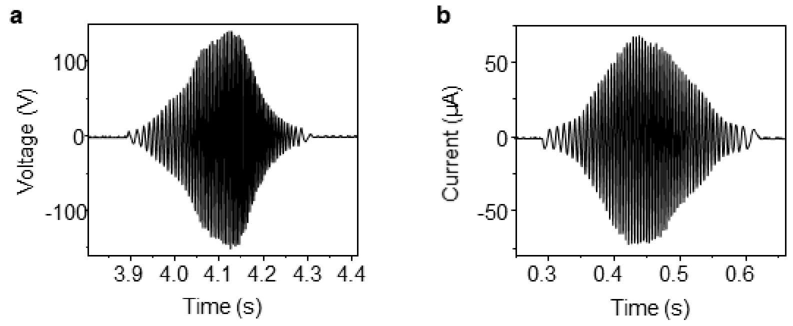

Supplementary Fig. 3 Raw voltage and current output of the optimized TEG module. Raw signal of (a) V_{OC} and (b) I_{SC} generated from the TEG module with 64 interdigitated grating units.

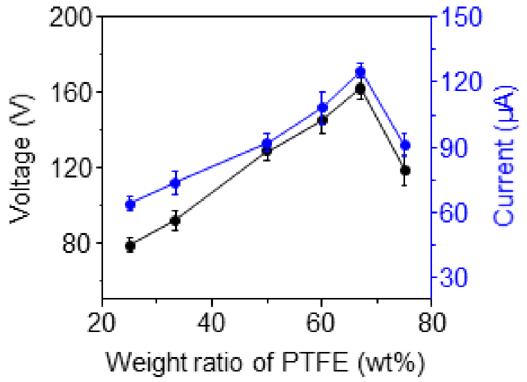

Supplementary Fig. 4 The output peak V_{OC} and I_{SC} of the TEG module with different ratios of PTFE with the binder.

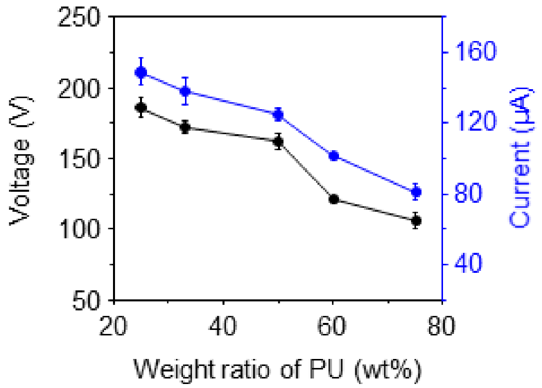

Supplementary Fig. 5 The output characteristic of the TEG module with different concentrations of PU for optimization of the positively charged layer.

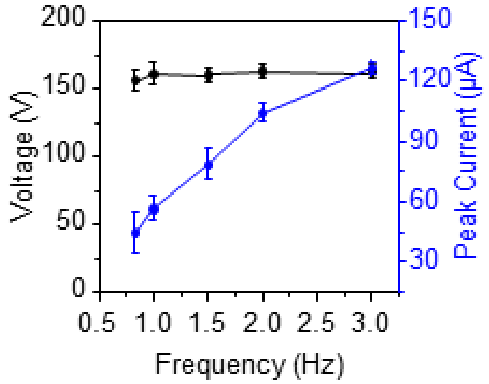

Supplementary Fig. 6 The peak voltage and peak current output of the TEG module with different frequencies ranging from 5/6 to 3 Hz of the TEG module.

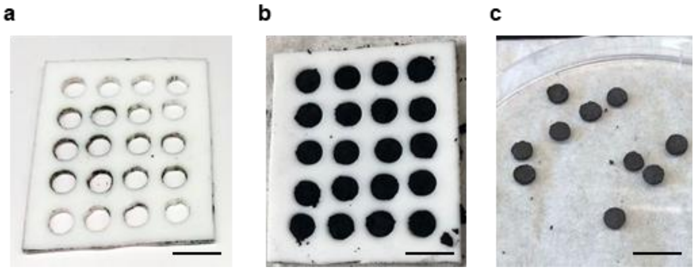

Supplementary Fig. 7 The fabrication of the CNT pellets for the BFC modules. (a) PTFE mold for CNT pellets. (b) PTFE mold filled by the CNT paste. (c) CNT pellets after drying. Scale bar, 1 cm.

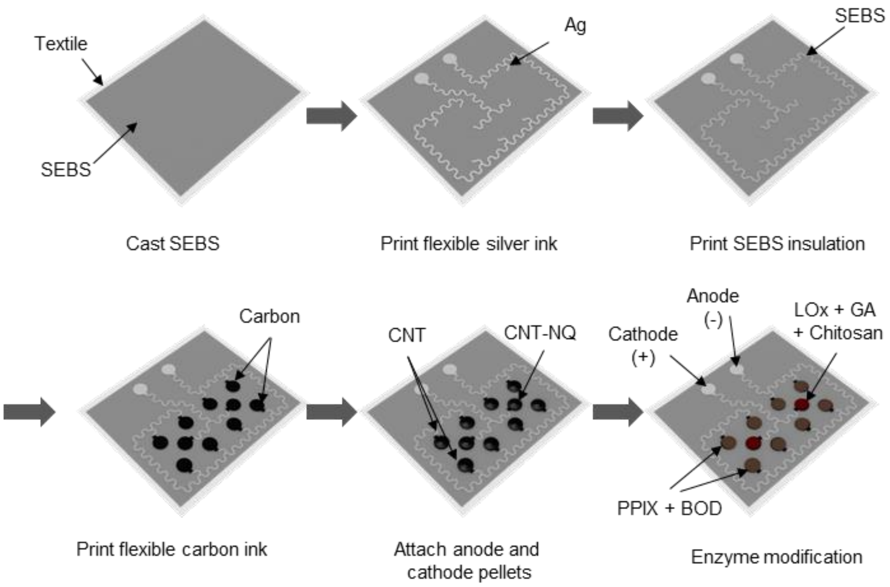

Supplementary Fig. 8 Illustration of the step-by-step assembly process of the BFC module. The current collector was printed layer-by-layer, followed by fixating the prepared CNT pellets and drop-casting of enzyme and other bonding solution.

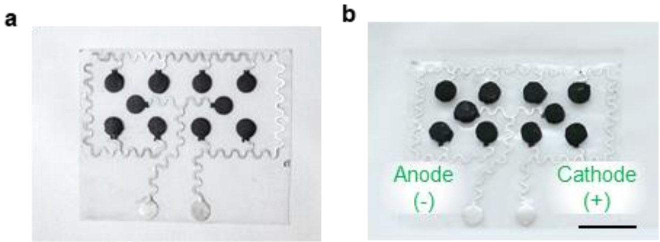

Supplementary Fig. 9 Photos of the BFC module. Top-down view of a fabricated BFC module (a) before and (b) after attaching the CNT pellets. Scale bar: 1 cm.

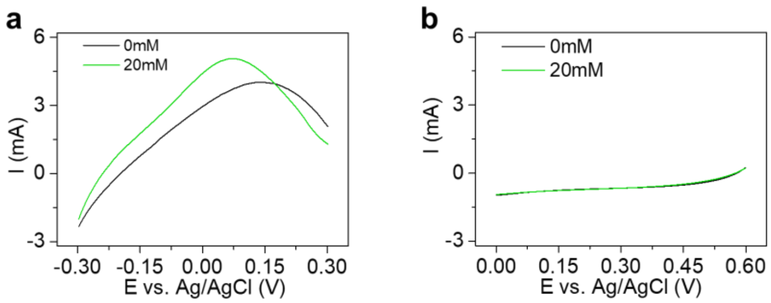

Supplementary Fig. 10 BFC single electrode performance with 0 mM and 20 mM lactate. (a) LSV on single anode electrode pellet. (b) LSV on single cathode electrode pellet. Scan rate: 5 mV s^{-1}.

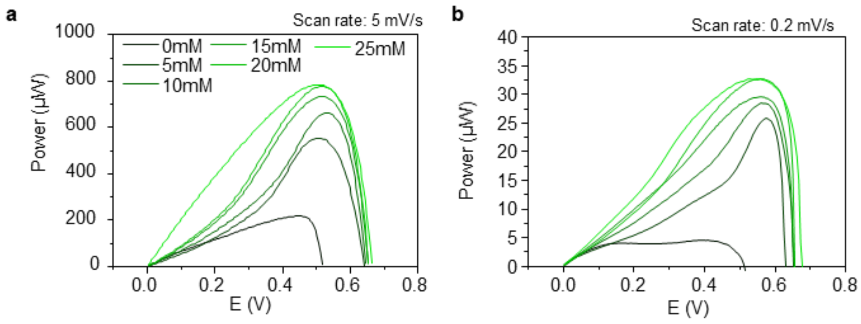

Supplementary Fig. 11 LSV characterization of the BFC module at different scan rates. The LSV polarization curve of one BFC module at different concentrations, characterized with a scan rate of (a) 5 mV s^{-1} and (b) 0.2 mV s^{-1}.

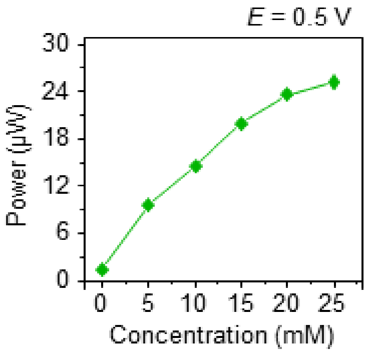

Supplementary Fig. 12 The power output of the BFC module discharged at 0.5 V with different lactate concentrations.

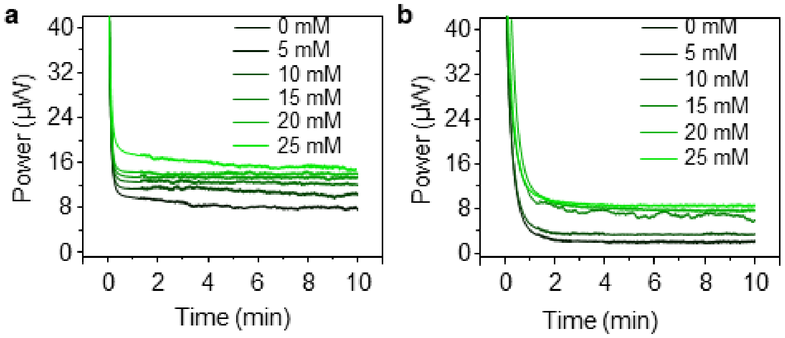

Supplementary Fig. 13 CA of the BFC module at different voltages. The power vs. time plot of a BFC module converted from the CA using Eq. 3 in the environment of 0, 5, 10, 15, 20, and 25 mM lactate at (a) 0.4 V and (b) 0.6 V.

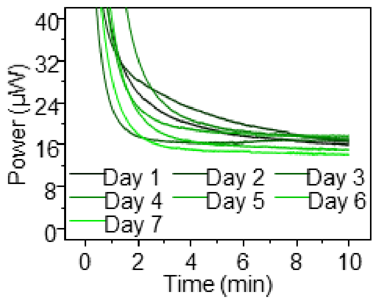

Supplementary Fig. 14 The CA of one BFC module scanned everyday within a week.

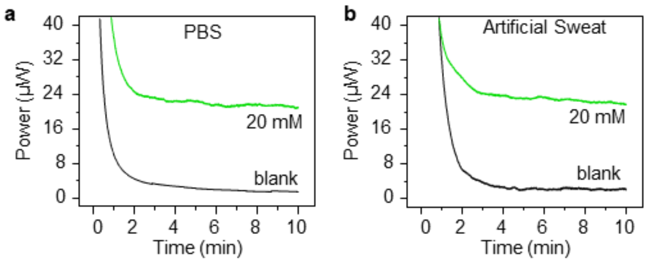

Supplementary Fig. 15 Performance characterization of the BFC in PBS and artificial sweat. The performance of the BFC module in (a) 0.5 M PBS and (b) artificial sweat composed of 85 mM of NaCl, 13 mM of KCl, and 16 mM of urea in 0.1 M PBS, with the lactate concentration of 0 mM and 20 mM.

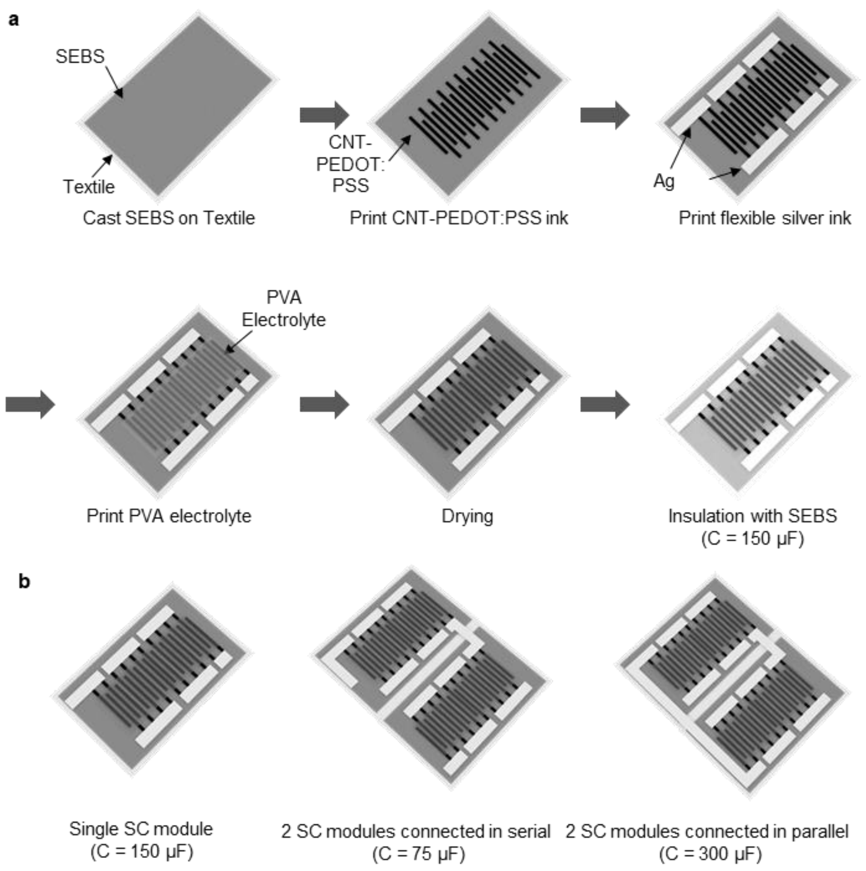

Supplementary Fig. 16 Illustration of the fabrication steps of the SC module. (a) Illustration of the layer-by-layer printing process of the SC modules. (b) Serial and parallel configurations of SC modules which result in a different amount of overall capacitance.

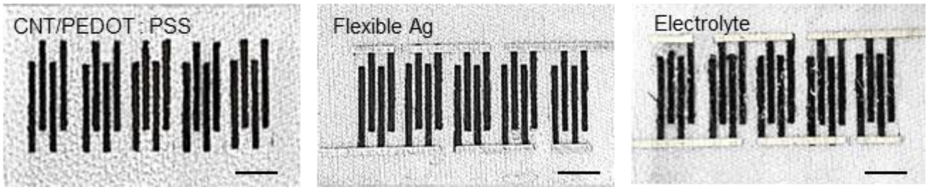

Supplementary Fig. 17 Top-down photo images of the printed SC module, where the electrodes, current collectors, and the electrolyte are printed layer by layer. Scale bar: 5 mm.

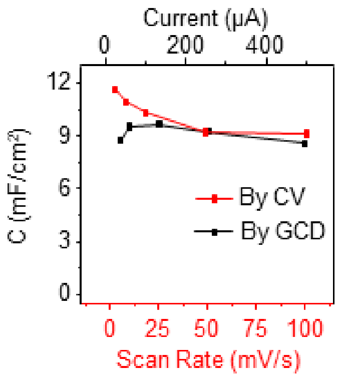

Supplementary Fig. 18 Summarized areal capacity of the printed SC characterized using different current in GCD and different scan rates in CV.

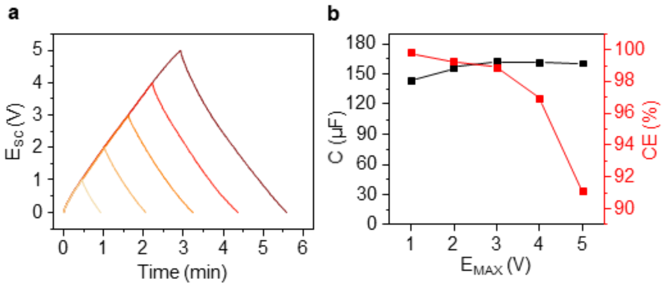

Supplementary Fig. 19 Charging efficiency characterization of the SC module. (a) GCD at 5 μA of one SC module between 0 V and the maximum voltage of 1, 2, 3, 4, and 5 V. (b) The summarized capacity and CE at different maximum voltage calculated using Eq. 5 and 6.

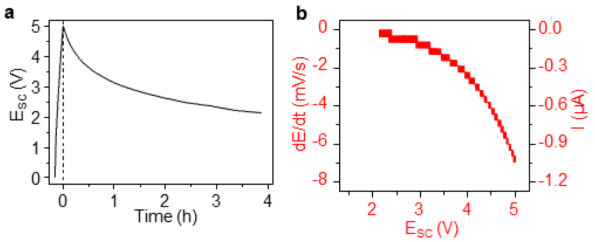

Supplementary Fig. 20 Self-discharge of the SC module. (a) Self-discharge of the 150 μF SC module charged to 5V in 4 hours. (b) The rate of discharging and corresponding self-discharge current at different states of charge of the SC module.

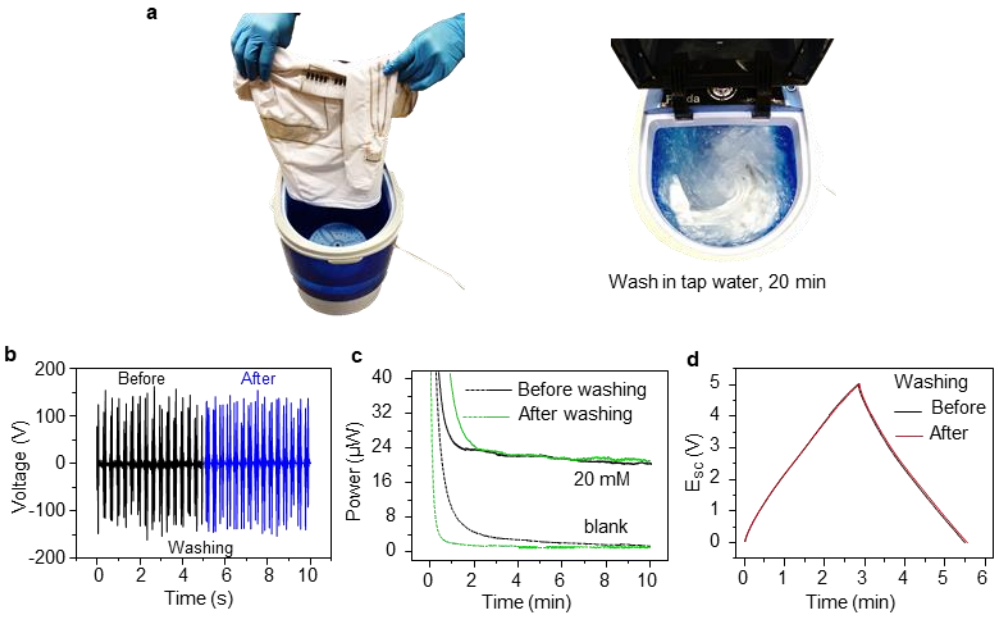

Supplementary Fig. 21 Component reliability test under washing tests. (a) Photo demonstrating the washing process of the integrated E-textile system. Room-temperature tap water without detergent was used to wash the shirt using a commercial washing machine for 20 min and dried under ambient environment. (b) The voltage output of the TEG module before and after the washing test. (c) The power output of the BFC module before and after the washing test in 0 mM and 20 mM lactate in PBS. (d) The GCD of the SC module before and after the washing test.

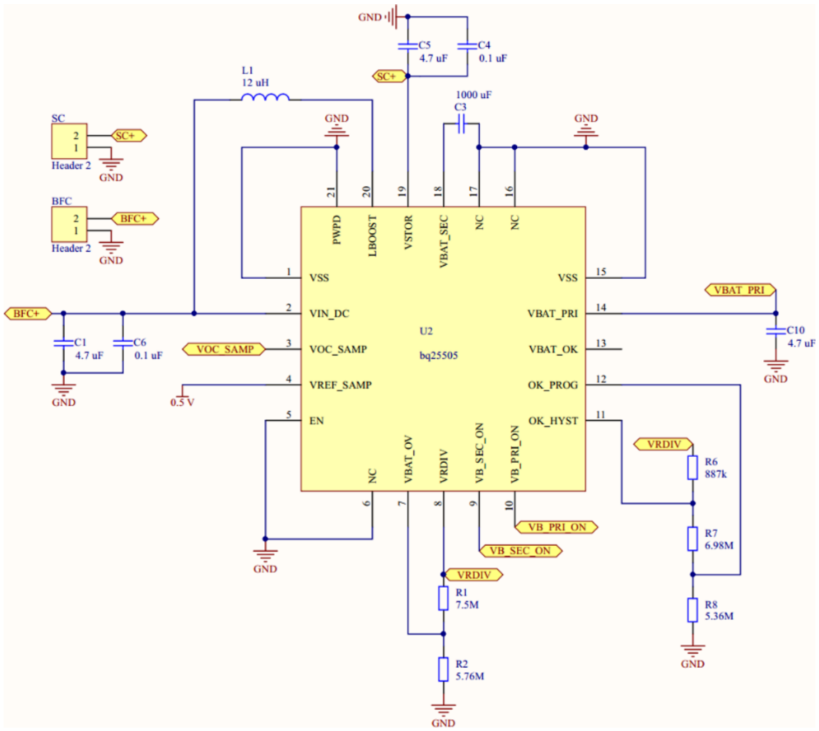

Supplementary Fig. 22 Schematics for the booster circuit system. The booster could extract power from BFC as low as 330 mV, with the desired potential adjustable via a reference voltage. Once the output voltage to the SC reaches 1.8 V, the booster could continuously harvest energy with high efficiency.

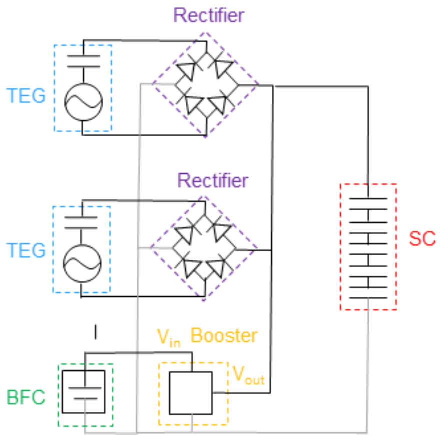

Supplementary Fig. 23 Circuit diagram for the integrated BFC and TEG energy harvesting modules, their corresponding regulating circuitry, and the SC energy storage modules. Gray lines: ground.

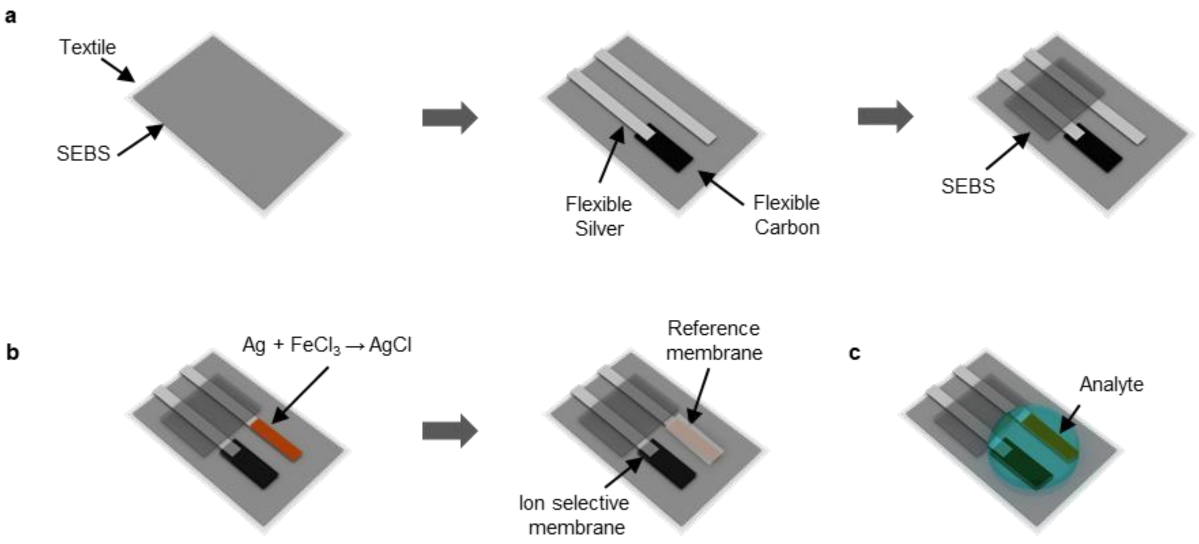

Supplementary Fig. 24 Illustration of the fabrication steps of the Na^+ sensor. (a) Layer-by-layer screen-printing of the electrode of the Na^+ sensor. (b) The functionalization of the Ag/AgCl reference electrode and the Na^+ ion-selective working electrode. (c) Fabricated Na^+ sensor in operation with the analyte.

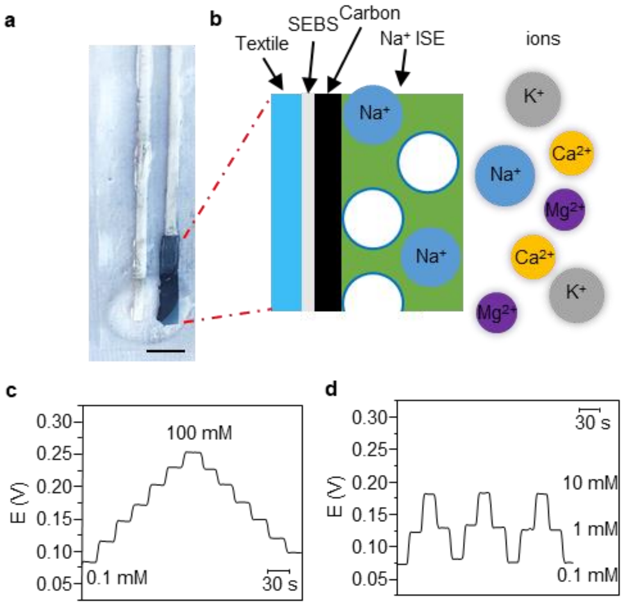

Supplementary Fig. 25 Sensing mechanism and characterization of the Na+ sensor. (a) Photo image of the printed and decorated Na^+ sensor. Scale bar, 2 mm. (b) Illustration of the sensing mechanism of the Na^+ ion-selective electrode (ISE). (c) The response of the Na^+ sensor to various Na^+ concentrations, ranging from 0.1 mM to 100 mM with the increment and decrement of one decade. (d) The reversible response of the ISE with 0.1 mM, 1 mM, and 10 mM of Na^+.

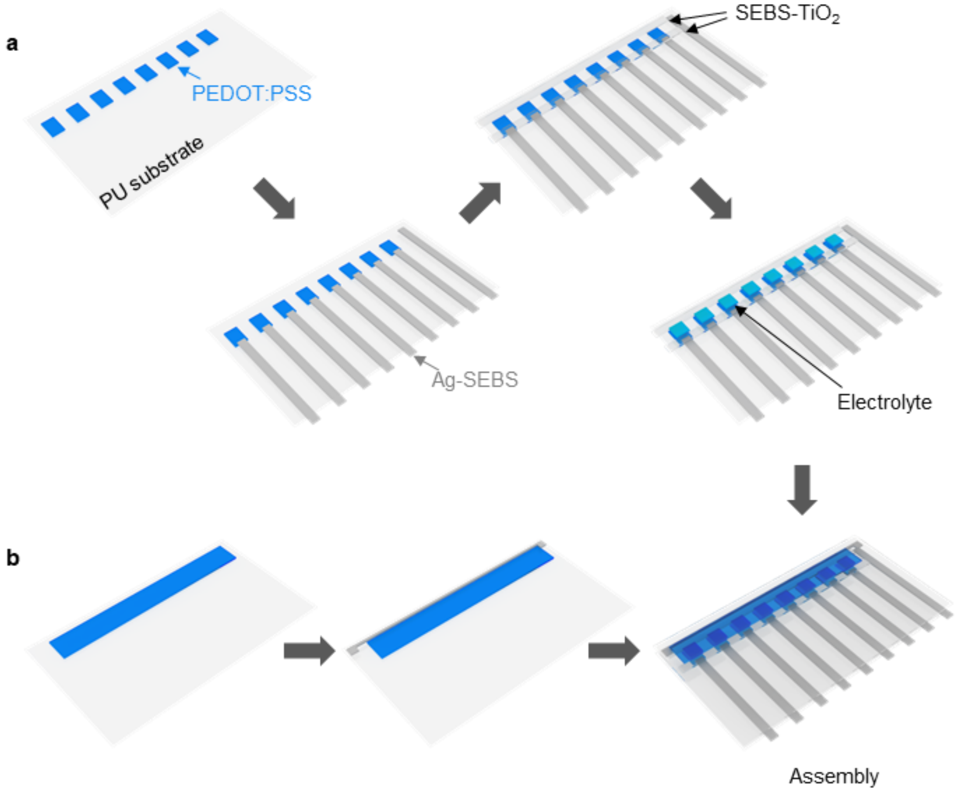

Supplementary Fig. 26 Fabrication steps of the ECD device. The layer-by-layer fabrication process of the ECD which is composed of the printing of (a) the back panel, (b) the front panel, and the assembly of the two.

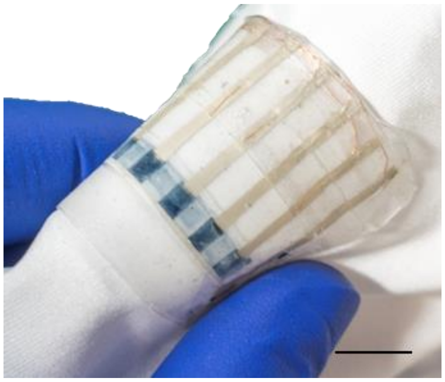

Supplementary Fig. 27 The photo image of the assembled ECD being bent 180°. Scale bar, 1 cm.

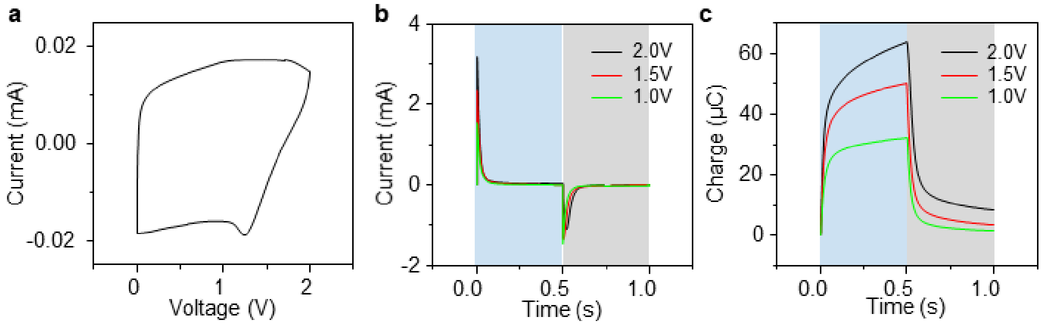

Supplementary Fig. 28 Electrochemical characterization of the ECD pixel. (a) CV of an individual ECD pixel with a scan rate of 0.1 V s^{-1}. The turn-on and the turn-off response of the ECD pixels with different turn-on voltages, in terms of (b) current and (c) charge. Blue region: set turn-on potential applied. Gray region: 0 V turn-off potential applied.

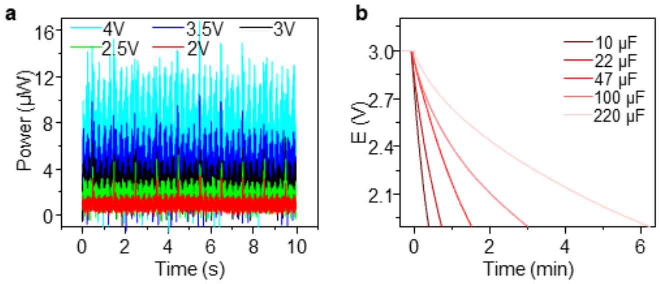

Supplementary Fig. 29 Characterizing the power consumption of the wrist watch. (a) the power consumption of the wristwatch when supplying a different level of voltage. (b) the discharge of capacitors charged to 3 V when connected to the wristwatch.

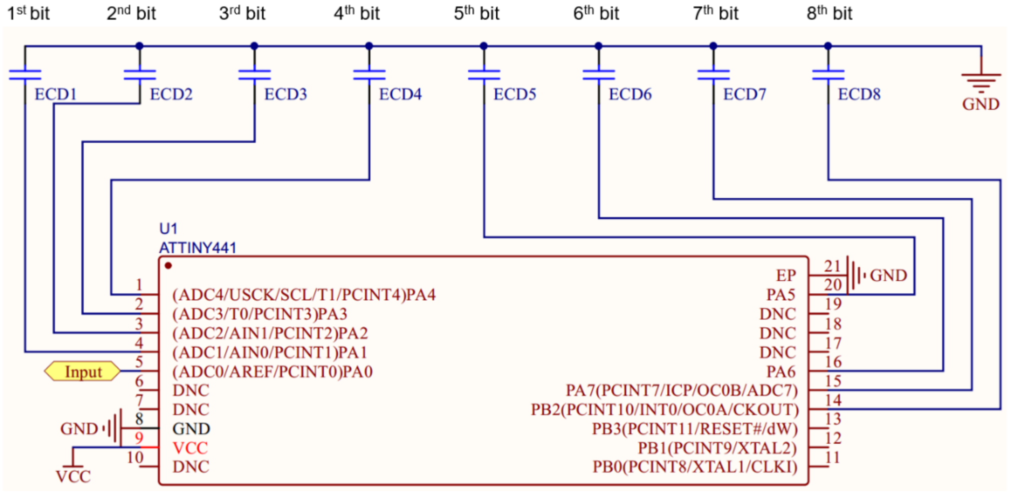

Supplementary Fig. 30 Schematics of the sensor-ECD system. The microcontroller (ATTINY441) is used to control the electrochromic displays. The input pin senses the potential level up to 0.32 V. The sensed voltage is visualized by an 8-bit electrochromic display, and each bit represents 0.04V.

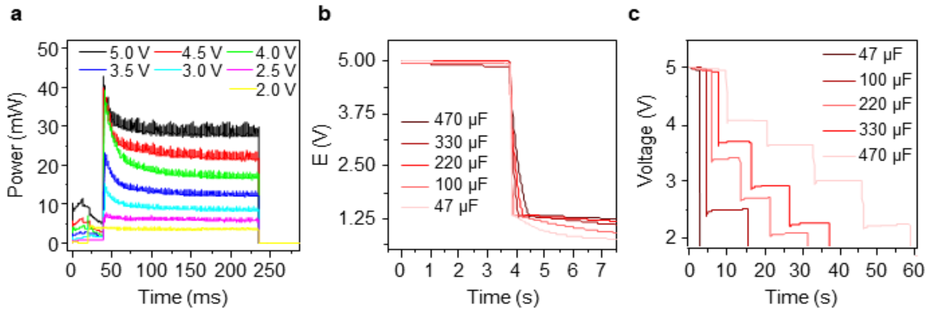

Supplementary Fig. 31 Characterizing the power consumption of the MCU. (a) The power consumption with different input voltages within a 200 ms operation session. (b) The continuous discharge profile of different capacitors charged to 5 V to power the microcontroller. (c) The repeated pulsed discharge profile of different capacitors charged to 5 V to power the microcontroller.

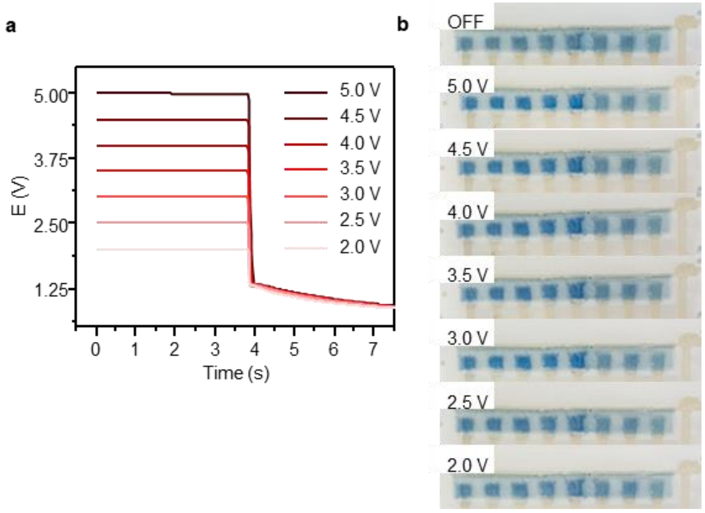

Supplementary Fig. 32 Analyzing the minimum energy needed to power the MCU-ECD sensing system. (a) Continuous discharge by the sensor-ECD system of the 75 μF SC module that was charged to a different state of charge and (b) the corresponding color change on the ECD.

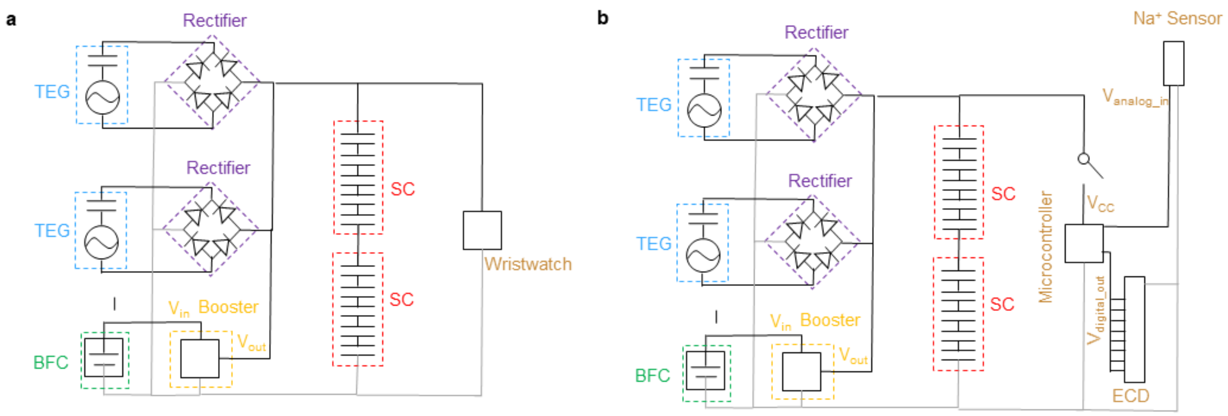

Supplementary Fig. 33 System diagram of the wristwatch and the MCU-ECD system. Circuit diagram for the multi-modular wearable microgrid system with applications including (a) a wristwatch operating continuously, and (b) a sensor-ECD system controlled by a microcontroller operating in pulsed sessions. Gray lines: ground.

Supplementary Movies

Supplementary Movie 1: Turning the ECD on and off

Supplementary Movie 2: The ECD responding to changing Na^+ concentrations

Supplementary References

1. Xie, Y. *et al.* Grating-Structured Freestanding Triboelectric-Layer Nanogenerator for Harvesting Mechanical Energy at 85% Total Conversion Efficiency. *Advanced Materials* **26**, 6599–6607 (2014).

2. Zhu, G. *et al.* Linear-grating triboelectric generator based on sliding electrification. *Nano Letters* **13**, 2282–2289 (2013).

3. Guo, H. *et al.* A Triboelectric Generator Based on Checker-Like Interdigital Electrodes with a Sandwiched PET Thin Film for Harvesting Sliding Energy in All Directions. *Advanced Energy Materials* **5**, 1400790 (2015).

4. Li, W. *et al.* Interdigitated Electrode-Based Triboelectric Sliding Sensor for Security Monitoring. *Advanced Materials Technologies* **3**, 1800189 (2018).

5. Kaur, N. & Pal, K. Triboelectric Nanogenerators for Mechanical Energy Harvesting. *Energy Technology* **6**, 958–997 (2018).

6. Bandodkar, A. J. *et al.* Soft, stretchable, high power density electronic skin-based biofuel cells for scavenging energy from human sweat. *Energy & Environmental Science* **10**, 1581–1589 (2017).

7. Bandodkar, A. J., Jeerapan, I., You, J. M., Nuñez-Flores, R. & Wang, J. Highly Stretchable Fully-Printed CNT-Based Electrochemical Sensors and Biofuel Cells: Combining Intrinsic and Design-Induced Stretchability. *Nano Letters* **16**, 721–727 (2016).

8. Chen, X. *et al.* Stretchable and Flexible Buckypaper-Based Lactate Biofuel Cell for Wearable Electronics. *Advanced Functional Materials* **29**, 1905785 (2019).

9. Jia, W., Valdés-Ramírez, G., Bandodkar, A. J., Windmiller, J. R. & Wang, J. Epidermal biofuel cells: Energy harvesting from human perspiration. *Angewandte Chemie - International Edition* **52**, 7233–7236 (2013).

10. Sakharov, D. A. *et al.* Relationship between lactate concentrations in active muscle sweat and whole blood. *Bulletin of Experimental Biology and Medicine* **150**, 83–85 (2010).

11. Derbyshire, P. J., Barr, H., Davis, F. & Higson, S. P. J. Lactate in human sweat: A critical review of research to the present day. *Journal of Physiological Sciences* **62**, (2012).

12. Tehrani, F. *et al.* Laser-Induced Graphene Composites for Printed, Stretchable, and Wearable Electronics. *Advanced Materials Technologies* **4**, 1900162 (2019).

13. Lv, J. *et al.* Sweat-based wearable energy harvesting-storage hybrid textile devices. *Energy & Environmental Science* **11**, 3431–3442 (2018).

14. Vosgueritchian, M., Lipomi, D. J. & Bao, Z. Highly Conductive and Transparent PEDOT:PSS Films with a Fluorosurfactant for Stretchable and Flexible Transparent Electrodes. *Advanced Functional Materials* **22**, 421–428 (2012).

15. Lipomi, D. J. *et al.* Electronic properties of transparent conductive films of PEDOT:PSS on stretchable substrates. *Chemistry of Materials* **24**, 373–382 (2012).

16. Zang, J., Cao, C., Feng, Y., Liu, J. & Zhao, X. Stretchable and High-Performance Supercapacitors with Crumpled Graphene Papers. *Scientific Reports* **4**, 1–7 (2014).

17. Xia, M., Nie, J., Zhang, Z., Lu, X. & Wang, Z. L. Suppressing self-discharge of supercapacitors via electrorheological effect of liquid crystals. *Nano Energy* **47**, 43–50 (2018).

18. Kowal, J. *et al.* Detailed analysis of the self-discharge of supercapacitors. *Journal of Power Sources* **196**, 573–579 (2011).

19. *TI bq25505 Datasheet*.

20. Gao, W. *et al.* Fully integrated wearable sensor arrays for multiplexed in situ perspiration analysis. *Nature* **529**, 509–514 (2016).

21. Emaminejad, S. *et al.* Autonomous sweat extraction and analysis applied to cystic fibrosis and glucose monitoring using a fully integrated wearable platform. *Proceedings of the National Academy of Sciences of the United States of America* **114**, 4625–4630 (2017).

22. Rich, M. *et al.* Circumventing Traditional Conditioning Protocols in Polymer Membrane-Based Ion-Selective Electrodes. *Analytical Chemistry* **88**, 8404–8408 (2016).

23. Andersson, P. *et al.* Active Matrix Displays Based on All-Organic Electrochemical Smart Pixels Printed on Paper. *Advanced Materials* **14**, 1460–1464 (2002).

24. Andersson Ersman, P., Kawahara, J. & Berggren, M. Printed passive matrix addressed electrochromic displays. **14**, 3371–3378 (2013).
